# Mitral valve prolapse and conduction disturbances: the forgotten association

**Published:** 2010-12

**Authors:** D’ALOIA ANTONIO, VIZZARDI ENRICO, ANTONIOLI ELENA, CHIARI ERMANNA, CURNIS ANTONIO, DEI CAS LIVIO

**Affiliations:** Department of Cardiology, University of Brescia, Italy; Department of Cardiology, University of Brescia, Italy; Department of Cardiology, University of Brescia, Italy; Department of Cardiology, University of Brescia, Italy; Department of Cardiology, University of Brescia, Italy; Department of Cardiology, University of Brescia, Italy

## Abstract

Various cardiac arrhythmias and conduction defects have been described in patients with mitral valve prolapse. We describe a case of a young woman affected by a mitral valve prolapse, involving the posterior mitral leaflet, with mild mitral regurgitation and an episode of syncope due to asystolia. It is hoped that this short communication will once again focus attention on the as yet unexplained association between mitral valve prolapse and various cardiac conduction disorders.

Various cardiac arrhythmias and conduction defects have been described in patients with mitral valve prolapse (MVP). 1–4 These include: sino–atrial and atrio–ventricular (AV) node dysfunction, prolongation of the QT interval, cases of refractory ventricular tachycardia and fibrillation, and dysautonomia.^5^–^9^ In symptomatic patients with mitral valve prolapse, infranodal conduction abnormalities as well as dual AV nodal pathways have been documented in electrophysiological studies.^10^

This is a case report of a 23–year–old woman who presented to our Department of Cardiology with an episode of syncope. Clinical examination revealed a grade II systolic murmur and the electrocardiogram showed a first–degree atrio–ventricular block (Fig. 1). Echocardiography demonstrated mitral valve prolapse involving the posterior leaflet, with mild mitral regurgitation (Figs 2, 3).

**Fig. 1:**
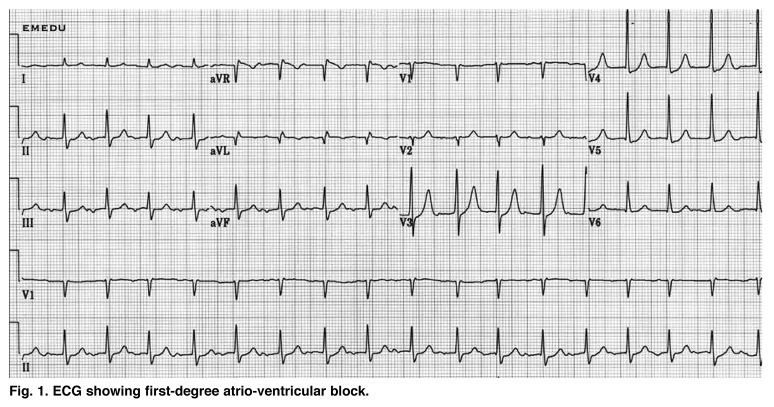
ECG showing first-degree atrio-ventricular block.

**Fig. 2:**
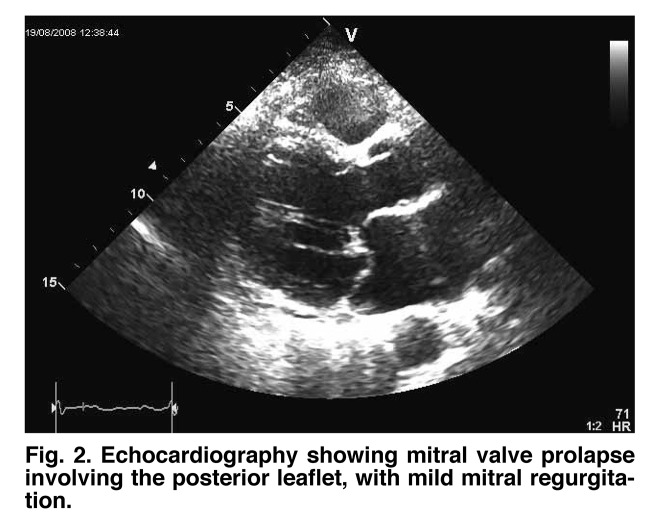
Echocardiography showing mitral valve prolapse involving the posterior leaflet, with mild mitral regurgitation.

**Fig. 3:**
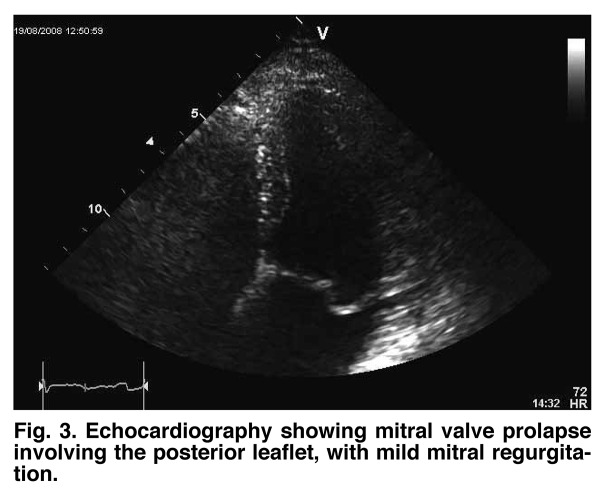
Echocardiography showing mitral valve prolapse involving the posterior leaflet, with mild mitral regurgitation.

Further diagnostic tests, including a chest X–ray, thyroid function tests and coronary angiography were all within normal limits. Telemetric electrocardiography revealed multiple episodes of asystole, the longest of which lasted for four seconds (Fig. 4). Following this, a permanent cardiac pacemaker was inserted.

**Fig. 4:**
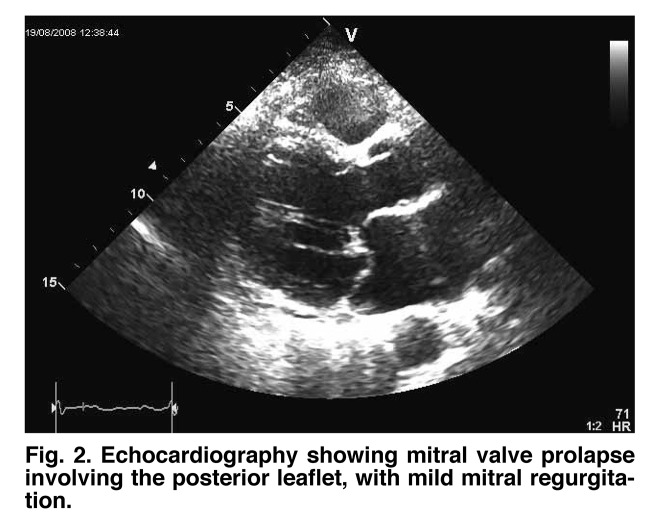
Telemetric ECG showing multiple episodes of asystole.

Tachyarrhythmias have been shown to represent the most frequent and potentially dangerous clinical manifestation of MVP and there is an association between mitral valve prolapse and sudden cardiac death.^11^–^13^

Atrio–ventricular conduction disturbances occur in mitral valve prolapse but the true mechanism(s) of arrhythmia is still unclear. In some patients with mitral valve prolapse, electro– physiological studies have demonstrated prolonged atrio–hisian intervals and/or abnormal responses to atrial pacing. In addition, a significant proportion of these patients had abnormalities of both sinus and atrio–ventricular node function, as well as distal His–Purkinje conduction abnormalities.

Atrio–ventricular block of all three degrees are well documented in mitral valve prolapse,^2^,^14^,^15^ and in some patients, it has been shown that atropine administration led to normalisation of atrio–ventricular conduction. This has led to speculation that in patients with mitral valve prolapse, an increased vagal tone is responsible for many of the conduction abnormalities.^15^ However, non–vagal causes are also possible and include the following: atrio–ventricular node developmental anomalies (as part of the floppy mitral valve) and/or compression of the atrioventricular nodal artery by the prolapsing leaflet, as the artery courses along the border of the mitral annulus.^16^

The prevalence of AV conduction disturbances among patients with mitral valve prolapse is probably higher than expected. It is hoped that this short communication will focus attention on the as yet unexplained association between mitral valve prolapse and various cardiac conduction disorders.
